# Prognostic impact of persistent versus transient sepsis-associated thrombocytopenia in multicohort data

**DOI:** 10.1016/j.isci.2025.114023

**Published:** 2025-11-11

**Authors:** Weimin Zhang, Xuping Cheng, Xufeng Cai, Zhongheng Zhang, Xuandong Jiang

**Affiliations:** 1 Intensive Care Unit, Affiliated Dongyang Hospital of Wenzhou Medical University, Dongyang, Zhejiang, China; 2 Department of Emergency Medicine, Sir Run Run Shaw Hospital, Zhejiang University School of Medicine, Hangzhou 310016, China; 3 Key Laboratory of Precision Medicine in Diagnosis and Monitoring Research of Zhejiang Province, Sir Run Run Shaw Hospital, Zhejiang University School of Medicine, Hangzhou, China; 4 School of Medicine, Shaoxing University, Shaoxing, China; 5 Longquan Industrial Innovation Research Institute, Lishui, China

**Keywords:** Cardiovascular medicine, Public health

## Abstract

Thrombocytopenia (TP) in patients with sepsis is associated with adverse outcomes. Previous research has predominantly focused on the severity of TP, while the prognostic impact of its duration remains insufficiently explored. This study aimed to investigate the association between the duration of sepsis-associated thrombocytopenia (SAT) and clinical outcomes. Over a 28-day observation period, patients with persistent TP (duration > 3 days) had significantly fewer ventilator-free days, intensive care unit (ICU)-free days, and hospital-free days (all *p* < 0.001) than those with transient TP (resolution within 1–3 days). Persistent TP was associated with significantly increased mortality (*p* < 0.001) and transfusion rates (*p* < 0.001). Multivariable logistic regression analysis revealed that transient TP was not significantly associated with in-hospital mortality (*p* > 0.05), whereas persistent TP remained an independent risk factor (*p* < 0.05). A 3-day cutoff to differentiate between transient and persistent SAT demonstrated significant clinical utility. The duration of TP offers additional, valuable prognostic information, potentially guiding risk stratification and therapeutic strategies.

## Highlights

• 3-day cutoff distinguishes transient vs. persistent sepsis thrombocytopenia
• Persistent SAT doubles mortality risk across three ICU cohorts
• Transient SAT shows no excess death versus no thrombocytopenia
• Duration-based SAT subtyping enables early risk stratification

## Graphical abstract

**Figure:**
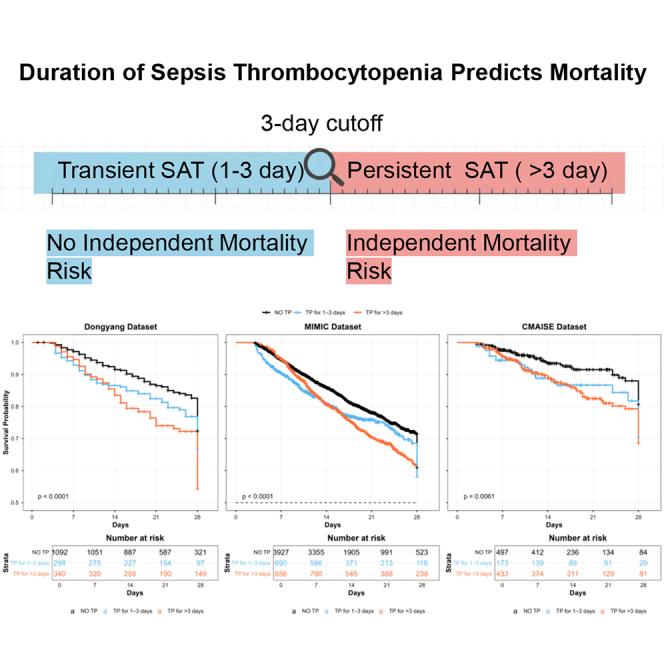

## Introduction

Sepsis, defined as life-threatening organ dysfunction caused by a dysregulated host response to infection, represents a major global health challenge, affecting approximately 20% of patients admitted to intensive care units (ICUs).^1^^,^^2^^,^^3^ Sepsis-associated thrombocytopenia (SAT) is a common complication in the ICU, with a reported incidence of 14.5%–55%.^4^^,^^5^ SAT significantly increases the mortality rate of patients.^6^^,^^7^ SAT is also linked to extended ICU stays, prolonged vasopressor treatment duration, increased blood transfusion rates, acute kidney injury, and other negative outcomes.^6^^,^^8^

Previous consensus guidelines and research on thrombocytopenia (TP) have focused on its severity.^9^^,^^10^ TP can be categorized into severe TP (platelet count ≤ 50 × 109/L) and non-severe TP (platelet count > 50 × 109/L.^11^^,^^12^ In addition, TP has also been divided into three groups—mild (platelet count < 100 × 109/L), moderate (platelet count ≤ 50 × 109/L), and severe (platelet count ≤2 0 × 109/L)—in some studies.^13^ Increased patient mortality is positively correlated with the extent of platelet count decrease.^14^^,^^15^

However, clinical observations suggest that the trajectory of platelet counts—their dynamic change over time—may harbor more comprehensive prognostic information than the nadir alone. TP can be further classified into two subcategories based on the trajectory of platelet counts. The first is transient TP, distinguished by an abrupt decline in the platelet count, followed by swift recovery. This type is frequently observed in patients who have undergone major surgical procedures; it is generally associated with a favorable prognosis. The second subcategory is persistent TP, characterized by prolonged periods of low platelet counts, gradual improvement in later stages, and a more unfavorable prognosis. Some patients with transient TP have short-term severe TP. The prognosis of patients with severe TP cannot be accurately estimated based solely on the absolute platelet count. Therefore, the temporal progression of alterations in platelet quantity is an additional crucial aspect in this matter.

Previous research has indicated that a decline in platelet production among patients in critical condition is linked to a rather unfavorable outlook.^16^ Meanwhile, the recovery of platelet count in patients with TP indicates improved chances of survival.^17^ A recent study that analyzed the progression of platelet count and prognosis in 436 patients with TP due to infectious shock reported a noteworthy correlation between TP duration and hospitalization and ICU mortality rates.^11^ However, to our knowledge, no rational cutoff values to distinguish between transient and persistent SAT currently exist. Early identification of transient and persistent SAT can help clinicians improve patient management by avoiding unnecessary examinations.

Therefore, this study aimed to investigate the association between the duration of TP and clinical outcomes of patients with SAT by utilizing data from three large, heterogeneous ICU patient cohorts, including both Chinese and international public databases. Specifically, we sought to identify and validate a clinically practical cutoff value for TP duration that can effectively distinguish between transient and persistent SAT subtypes. The findings are intended to provide an evidence base for a more refined classification of SAT, facilitate earlier and more accurate prognostic assessment, and potentially inform the development of individualized therapeutic strategies.

## Results

### Baseline characteristics of patients

**Figure 1:**
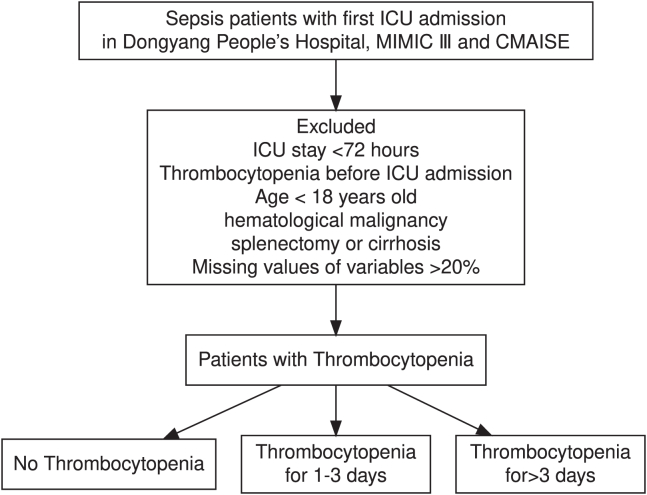
Flowchart of patient selection

A total of 1,730 patients from Dongyang People’s Hospital, 5,473 from Medical Information Mart for Intensive Care III (MIMIC-III, version 1.4), and 1,103 from Chinese Multi-omics Advances In Sepsis 1.5 Consortium (CMAISE V1.5) were included in the final analysis (Figure 1 for patient selection flowchart).

In the Dongyang cohort (Table 1), 1,092 (63.1%) patients had no TP, 298 (17.2%) developed transient TP (1–3 days), and 340 (19.7%) had persistent TP (>3 days). Overall, 62.5% (1,082/1,730) of the patients were male, with no significant differences in sex distribution across the three groups (*p* = 0.197). Patients in the persistent TP group were significantly older (mean age: 66.9 ± 14.5 years) than those in the transient TP (63.7 ± 16.1 years) and no TP groups (62.5 ± 16.0 years) (*p* < 0.001). Disease severity upon admission, indicated by Acute Physiology and Chronic Health Evaluation II (mean: 22.0 ± 7.4 vs. 21.0 ± 6.6 vs. 19.1 ± 6.3, respectively; *p* < 0.001) and Sequential Organ Failure Assessment scores (mean: 8.4 ± 3.4 vs. 7.0 ± 3.0 vs. 5.8 ± 2.7, respectively; *p* < 0.001), was significantly higher in the persistent TP group. Similarly, procalcitonin (PCT) levels (median 3.0 [interquartile rage: 0.61, 22.16] μg/L), lactate levels (mean 3.3 ± 2.1 mmol/L), and markers of coagulopathy (prothrombin time [PT], international normalized ratio [INR], activated partial thromboplastin time [APTT], and D-dimer) and renal dysfunction (creatinine and urea) on admission were significantly more deranged in the persistent TP group than in the other two groups (all *p* < 0.001). Platelet count (mean: 102.9 ± 55.4×10^9^/L) and hemoglobin level on admission were lower (mean: 104 ± 25.7 g/L) in the persistent TP group than in the other groups (both *p* < 0.001). The mean platelet volume was significantly higher in both the transient TP (10.0 ± 1.4×10^15^/L) and persistent TP groups (10.0 ± 1.5×10^15^/L) than in the no TP group (9.5 ± 1.2×10^15^/L) (*p* < 0.001).

**Table 1:** Comparisons of baseline characteristics and outcomes between patients with and without thrombocytopenia

Variables	Total (*N* = 1,730)<	No TP (*n* = 1,092)	TP 1–3 days (*n* = 298)	TP > 3 days (*n* = 340)	*p* value
Age, (years)	63.5 ± 15.8	62.5 ± 16	63.7 ± 16.1	66.9 ± 14.5	<0.001
Sex, men (%)	1,082 (62.5)	671 (61.4)	200 (67.1)	211 (62.1)	0.197
Hypertension (%)	875 (50.6)	627 (57.4)	109 (36.6)	139 (40.9)	<0.001
Diabetes (%)	269 (15.5)	183 (16.8)	40 (13.4)	46 (13.5)	0.192
COPD (%)	205 (11.8)	137 (12.5)	23 (7.7)	45 (13.2)	0.05
Site of infection (%)	–	–	–	–	<0.001
Thorax	1,122 (64.9)	781 (71.5)	175 (58.7)	166 (48.8)	–
Abdomen	190 (11)	86 (7.9)	46 (15.4)	58 (17.1)	–
Blood	119 (6.9)	52 (4.8)	28 (9.4)	39 (11.5)	–
Soft tissue	59 (3.4)	28 (2.6)	14 (4.7)	17 (5)	–
UTI	129 (7.5)	72 (6.6)	19 (6.4)	38 (11.2)	–
Other	111 (6.4)	73 (6.7)	16 (5.4)	22 (6.5)	–
RRT (%)	148 (8.6)	54 (4.9)	20 (6.7)	74 (21.8)	<0.001
APACHE-II score	20 ± 6.7	19.1 ± 6.3	21 ± 6.6	22 ± 7.4	<0.001
SOFA score	6.5 ± 3.1	5.8 ± 2.7	7 ± 3	8.4 ± 3.4	<0.001
Antiplatelet drug use (%)	221 (12.8)	165 (15.1)	25 (8.4)	31 (9.1)	<0.001
Vasopressor use (%)	1,094 (63.2)	615 (56.3)	209 (70.1)	270 (79.4)	<0.001
Severe TP (%)	207 (12)	0 (0)	30 (10.1)	177 (52.1)	<0.001
Duration of TP (days)	0 (0, 1.95)	0 (0, 0)	1 (0, 1.99)	5.42 (4, 9.92)	<0.001

**Laboratory indexes on first ICU admission day**					

Red blood cell count (×10^9^/L)	3.7 ± 0.7	3.8 ± 0.6	3.6 ± 0.7	3.4 ± 0.8	<0.001
White blood cell count (×10^9^/L)	12.3 ± 5.3	12.3 ± 4.8	12.7 ± 6	12.1 ± 6	0.387
Lymphocyte count (×10^9^/L)	0.9 ± 0.8	1 ± 0.7	1 ± 1	0.8 ± 0.7	0.007
Platelet count (×10^9^/L)	169.8 ± 74.6	199.2 ± 66.4	138.4 ± 58.4	102.9 ± 55.4	<0.001
Mean platelet volume (×10^15^/L)	9.7 ± 1.3	9.5 ± 1.2	10 ± 1.4	10 ± 1.5	<0.001
Plateletcrit (%)	0.2 ± 0.1	0.2 ± 0.1	0.2 ± 0.1	0.1 ± 0.1	<0.001
Platelet distribution width (×10^15^/L)	16.2 (15.9, 16.5)	16.1 (15.8, 16.4)	16.3 (15.95, 16.6)	16.3 (16, 16.7)	<0.001
Hemoglobin (g/L)	111.1 ± 21.8	114.1 ± 19.8	107.8 ± 22	104 ± 25.7	<0.001
C-reactive protein (mg/L)	71.85 (23.42, 140.57)	62.63 (20.59, 135.45)	89.99 (43.81, 157.85)	79.7 (25.53, 145.69)	<0.001
Procalcitonin (ug/L)	0.66 (0.17, 3.29)	0.39 (0.12, 1.4)	1.4 (0.4, 6.79)	3 (0.61, 22.16)	<0.001
pH	7.4 ± 0.1	7.4 ± 0.1	7.4 ± 0.1	7.4 ± 0.1	<0.001
PCO_2_ (mmHg)	35.2 ± 6.8	35.6 ± 6.5	34.8 ± 7.2	34.6 ± 7.3	0.023
PO_2_ (mmHg)	145.8 ± 54.1	145.9 ± 53.1	145.7 ± 55.5	145.5 ± 56.1	0.995
Bicarbonate (mmol/L)	21.3 ± 3.8	22 ± 3.5	20.7 ± 4	19.7 ± 4.2	<0.001
Lactate (mmol/L)	2.6 ± 1.7	2.3 ± 1.4	2.8 ± 1.7	3.3 ± 2.1	<0.001
Prothrombin time (s)	14.8 (13.8, 16)	14.4 (13.6, 15.3)	15.25 (14.4, 16.4)	16.1 (14.8, 18.5)	<0.001
International normalized ratio	1.17 (1.08, 1.3)	1.13 (1.06, 1.22)	1.22 (1.13, 1.34)	1.3 (1.18, 1.54)	<0.001
APTT (s)	39.2 (35.4, 44.6)	37.9 (34.7, 42.5)	40.6 (36.52, 45.5)	43.2 (38.38, 52.73)	<0.001
D-dimer (μg/L)	3.79 (1.74, 9.49)	2.87 (1.43, 6.72)	5.08 (2.27, 12.67)	7.27 (2.83, 16)	<0.001
Creatinine (mmol/L)	73 (55, 102)	68 (52, 89)	75.5 (56, 105.75)	94 (69, 158.5)	<0.001
Urea (mmol/L)	8.4 ± 5.2	7.5 ± 4.5	8.6 ± 5.1	11.1 ± 6	<0.001

**Vital signs on first ICU admission day**					

Systolic pressure (mmHg)	135.3 ± 29.7	140.1 ± 29.4	130.9 ± 28.9	123.7 ± 27.4	<0.001
Diastolic pressure (mmHg)	74.5 ± 15.3	76.7 ± 15.1	71.5 ± 14.6	70.1 ± 15	<0.001
Temperature (°C)	36.8 ± 1	36.9 ± 1	36.8 ± 1	36.6 ± 1.2	<0.001
Heart rate (bpm)	89.5 ± 21	86.7 ± 19.9	93.2 ± 22.2	95.4 ± 21.7	<0.001
Respiratory rate (breaths per min)	14 (12, 18)	14 (12, 17)	14 (12, 18)	14 (12, 20)	0.054

Abbreviations: TP, thrombocytopenia; COPD, chronic obstructive pulmonary disease; UTI, urinary tract infection; RRT, renal replacement therapy; APACHE, Acute Physiology and Chronic Health Evaluation; SOFA, Sequential Organ Failure Assessment; ICU, intensive care unit; PCO2, partial pressure of carbon dioxide; PO2, partial pressure of oxygen; APTT, activated partial thromboplastin time.

Regarding treatments, patients with persistent TP exhibited significantly increased rates of renal replacement therapy (21.8% vs. 6.7% vs. 4.9%; *p* < 0.001) and vasopressor use (79.4% vs. 70.1% vs. 56.3%; *p* < 0.001). The incidence of severe TP (platelet count <50×10^9^/L) was also highest in the persistent TP group (52.1% vs. 10.1% vs. 0%; *p* < 0.001). Detailed baseline characteristics are presented in Table 1.

Baseline characteristics and intergroup comparisons in the MIMIC-III and CMAISE1.5 cohorts exhibited similar trends (Tables S2 and S3). The median TP duration for SAT patients in the MIMIC-III cohort was 3.5 days.

### Clinical outcomes

In the Dongyang cohort (Table 2), in-hospital mortality was 35.6% (121/340) in the persistent TP group, significantly higher than 23.5% (70/298) in the transient TP group and 17.3% (189/1,092) in the no TP group (*p* < 0.001). The overall in-hospital mortality among patients with SAT (transient or persistent TP) was 29.9% (191/638).

**Table 2:** Comparisons of outcomes between patients with and without thrombocytopenia

Variables	Total (*N* = 1,730)	No TP (*n* = 1,092)	TP 1–3 days (*n* = 298)	TP > 3 days (*n* = 340)	*P* value
Ventilator-free days	20.29 (0.23, 26.33)	21.47 (10.19, 26.35)	19.69 (0, 25.89)	16.03 (0, 26.44)	<0.001
ICU-free days	15.05 (0, 21.71)	16.15 (4.27, 22.18)	14.03 (0, 21.47)	8.11 (0, 19.83)	<0.001
Hospital-free days	1 (0, 10)	3 (0, 10)	0 (0, 10)	0 (0, 6)	<0.001
Hospital mortality (%)	380 (22)	189 (17.3)	70 (23.5)	121 (35.6)	<0.001
Cost (×103 yuan)	70.63 (46.66, 102.22)	66.04 (45.47, 91.48)	71.76 (44.1, 109.22)	91.55 (56.22, 137.93)	<0.001
RBC transfusion (mL)	0 (0, 675)	0 (0, 400)	400 (0, 900)	700 (0, 1,600)	<0.001
FFP transfusion (mL)	0 (0, 440)	0 (0, 270)	235 (0, 715)	470 (0, 1,460)	<0.001
Platelet transfusion (%)	106 (6.1)	1 (0.1)	9 (3)	96 (28.2)	<0.001
RBC and FFP transfusion, n (%)	475 (27.5)	170 (15.6)	117 (39.3)	188 (55.3)	<0.001
FFP and platelet transfusion, n (%)	98 (5.7)	1 (0.1)	9 (3)	88 (25.9)	<0.001
RBC and platelet transfusion, n (%)	84 (4.9)	1 (0.1)	9 (3)	74 (21.8)	<0.001
RBC, FFP, and platelet transfusion, n (%)	79 (4.6)	1 (0.1)	9 (3)	69 (20.3)	<0.001

Abbreviations: TP, thrombocytopenia; ICU, intensive care unit; RBC, red blood cell; FFP, fresh frozen plasma.

Patients with persistent TP had significantly fewer 28-day (median: 16.03 days), ICU-free days (median: 8.11 days), and hospital-free days (median: 0 days) than those with no TP or transient TP (all *p* < 0.001). Hospitalization costs were significantly higher in the persistent TP group (median: 91.55×10^3^ CNY; *p* < 0.001). Furthermore, the persistent TP group required significantly greater volumes of red blood cell and fresh frozen plasma transfusions and had a higher proportion of patients receiving platelet transfusions than the other groups (28.2% vs. 3.0% vs. 0.1%; all *p* < 0.001 compared to transient and no TP groups, respectively). Analysis of transfusion triggers in the Dongyang cohort revealed that the median pre-transfusion platelet count was 30.0×10^9^/L, median pre-transfusion hemoglobin level was 8.2 g/dL, and median pre-transfusion INR was 1.35 (PT: 15.7 s) (Figure S2).

The clinical outcomes in the MIMIC-III dataset (overall SAT in-hospital mortality: 32.0%) and CMAISE1.5 cohort mirrored these trends (Tables S2 and S3).

### Association between TP duration and in-hospital mortality

After multivariable adjustment for potential confounders (Table 3), persistent TP was consistently associated with increased in-hospital mortality compared to that in the no TP group across all the three cohorts—Dongyang (adjusted odds ratio [aOR]: 1.94, 95% confidence interval [CI]: 1.37–2.76), MIMIC-III (aOR: 1.69, 95% CI: 1.40–2.04), and CMAISE1.5 (aOR: 1.90, 95% CI: 1.04–3.52). In contrast, transient TP was not significantly associated with in-hospital mortality in any cohort: Dongyang (aOR: 1.36, 95% CI: 0.95–1.92), MIMIC-III (aOR: 1.21, 95% CI: 0.98–1.48), and CMAISE1.5 (aOR: 1.62, 95% CI: 0.86–3.01).

**Table 3:** Multivariable logistic regression analysis of hospital mortality risk associated with thrombocytopenia duration

Adjusted OR (95% CI)			
Duration of thrombocytopenia	Dongyang	MIMIC Ⅲ	CMAISE
None	1.00 reference	1.00 reference	1.00 reference
1–3 days	1.33 (0.94–1.88)	1.21 (0.98–1.48)	1.62 (0.86–3.01)
>3 days	1.90 (1.35–2.68)	1.69 (1.40–2.04)	1.90 (1.04–3.52)

Abbreviations: 95% CI, 95% confidence interval; OR, odds ratio. Dongyang model: adjusted for age, sex, Acute Physiology and Chronic Health Evaluation II score, mechanical ventilation, chronic obstructive pulmonary disease, pH, lactate, surgery, renal replacement therapy, antiplatelet drug use, transfusion of blood products, and prothrombin time. MIMIC-III models: adjusted for age, sex, Sequential Organ Failure Assessment score, hypertension, chronic obstructive pulmonary disease, vasopressor use, prothrombin time, creatinine, pH, lactate, and surgery; CMAISE model: adjusted for age, sex, hypertension, diabetes, SOFA score, lactate, pH, renal replacement therapy, platelet count, mechanical ventilation, and chronic obstructive pulmonary disease.

**Figure 2:**
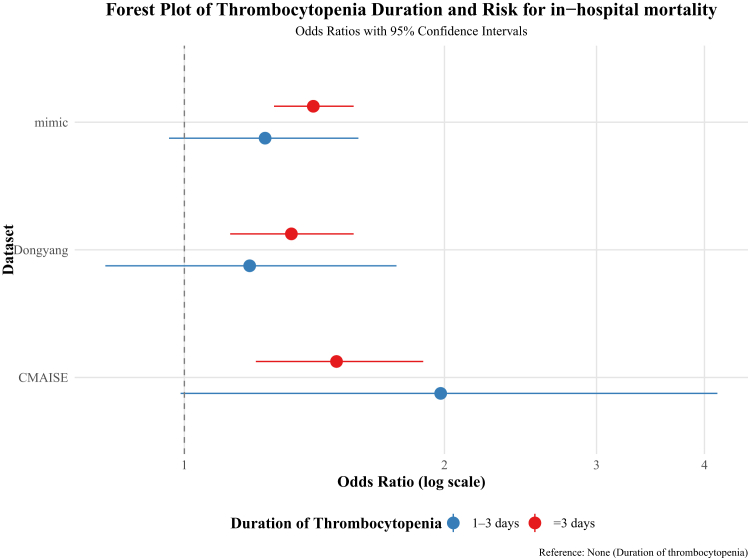
Forest plots of odds ratios for in-hospital mortality after propensity score matching

Propensity score matching (PSM) analyses (Table 4, Figure 2) corroborated these findings. After 1:1 matching, in-hospital mortality did not significantly differ between the transient TP group and the matched no TP group in any cohort (Dongyang: 23.5% vs. 20.5%, *p* = 0.429; MIMIC-III: 24.9% vs. 21.2%, *p* = 0.110; CMAISE1.5: 13.9% vs. 7.5%, *p* = 0.082). However, the persistent TP group consistently demonstrated significantly higher in-hospital mortality than its matched no TP counterparts (Dongyang: 35.6% vs. 23.8%, *p* = 0.001; MIMIC-III: 37.7% vs. 23.4%, *p* < 0.001; CMAISE1.5: 15.2% vs. 7.4%, *p* < 0.001).

**Table 4:** Propensity score-matched cohort outcomes by group

Dataset	Analysis type	Total in matched analysis (n)	1:1	Outcome (mortality, %)	*p* value
CMAISE	no TP: TP 1–3 days	346	173:173	7.5% vs. 13.9%	0.082
	no TP: TP > 3 days	866	433:433	7.4% vs. 15.2%	<0.001
Dongyang	no TP: TP 1–3 days	596	298:298	20.5% vs. 23.5%	0.429
	no TP: TP > 3 days	680	340:340	23.8% vs. 35.6%	0.001
MIMIC III	no TP: TP 1–3 days	1,380	690:690	21.2% vs. 24.9%	0.110
	no TP: TP > 3 days	1,712	856:856	23.4% vs. 37.7%	<0.001

**Figure 3:**
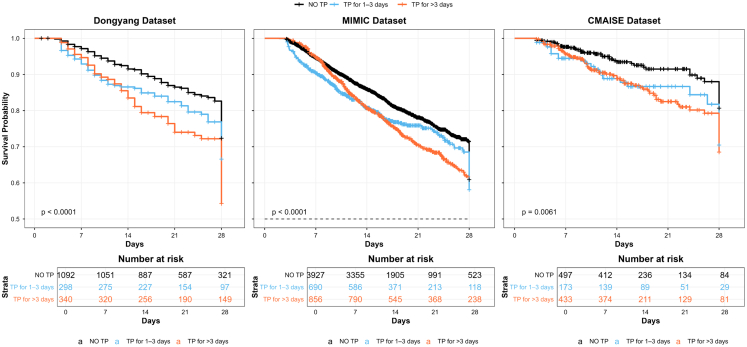
Kaplan-Meier survival curves for patients grouped by duration of thrombocytopenia

Kaplan-Meier survival curves (Figure 3) illustrated significantly lower in-hospital survival rates for patients with persistent SAT than for those with transient SAT or no TP across all three datasets (log rank test, all *p* < 0.05).

### Sensitivity analysis

Sensitivity analyses using a 2-day cutoff for TP duration (Table S4) in the Dongyang and MIMIC-III datasets yielded results consistent with those of the primary analysis—adjusted transient TP (1–2 days) was not significantly associated with in-hospital mortality, while persistent TP (>2 days) remained an independent risk factor. Conversely, when a 4-day cutoff was applied (Table S5), the adjusted risk of in-hospital mortality for transient TP (1–4 days) started to approach or achieve statistical significance relative to that in the no TP group, reinforcing 3 days as a more optimal discriminatory threshold. X-tile analysis further supported the use of 3 days as an appropriate cutoff for prognostic differentiation (Figure S1). Furthermore, the sensitivity analysis including patients with an ICU stay ≥24 h to account for early mortality yielded results consistent with the primary analysis, showing that persistent TP remained a significant risk factor for in-hospital mortality (Table S6).

## Discussion

We systematically evaluated the relationship between the duration of TP and clinical prognosis in patients with sepsis using data from three large, heterogeneous ICU databases. Our principal finding is that a TP duration exceeding 3 days can effectively differentiate SAT into two distinct prognostic subtypes—transient and persistent SAT. Persistent SAT was significantly associated with poorer clinical outcomes, including higher in-hospital mortality; fewer ventilator-free, ICU-free, and hospital-free days; and higher transfusion requirements than those in transient SAT. Critically, after rigorous adjustment for multiple potential confounders using multivariable logistic regression and propensity score matching, persistent SAT remained an independent risk factor for in-hospital mortality, whereas transient SAT showed no significant independent association with this outcome. This finding underscores the clinical importance of distinguishing these SAT subtypes and suggests that patients with persistent SAT may require closer monitoring, more in-depth etiological investigation, and potentially more aggressive intervention strategies.

Previous research and consensus guidelines on TP in sepsis have predominantly emphasized the prognostic significance of platelet count nadir or overall severity of TP.^10^^,^^14^^,^^15^ While a profound decrease in platelet count undoubtedly signals an adverse prognosis, our study showed that the dynamic recovery process—specifically, the duration of TP—is of substantial and independent prognostic value. We observed that some patients, even those experiencing a sharp decline in platelet counts, may have a favorable prognosis comparable to that of patients without TP if platelet recovery is prompt (i.e., transient TP). This finding aligns with earlier findings, namely that successful platelet count restoration is associated with improved survival.^16^^,^^17^ By proposing and validating a 3-day threshold, our study offers a clinically operational definition for “rapid recovery,” thus addressing a notable heterogeneity in the literature, where definitions of TP duration have shown variations; for instance, Zhou et al. reported that TP persisting for 7 days was linked to adverse outcomes in ICU sepsis patients, while Schupp et al. observed platelet count recovery commencing around day 5.^18^^,^^19^ Our observed median TP duration of approximately 3.2–3.5 days, supported by sensitivity and X-tile analyses validating the 3-day cutoff, suggests the potential for earlier identification of patients at high risk. Although more complex methodologies such as latent growth mixture models, as employed by Li et al. and Wang et al., have also identified prolonged low platelet counts as detrimental, the 3-day classification proposed herein offers a simpler, more readily applicable tool for routine clinical practice.^20^^,^^21^

The association between persistent SAT and adverse outcomes likely involves multifaceted pathophysiological mechanisms. First, persistent TP may signify an uncontrolled or sustained host inflammatory response to infection. In our cohorts, patients with persistent SAT exhibited increased admission PCT levels and more pronounced coagulation abnormalities (e.g., elevated PT, APTT, and D-dimer), indicative of a more severe and enduring inflammatory state and endothelial injury.^22^^,^^23^ Platelets are pivotal not only in hemostasis but also as key modulators of inflammation and immunity. Sustained inflammatory stimuli can disrupt the equilibrium between increased platelet consumption (e.g., aggregation at sites of endothelial damage and microthrombi formation) and suppressed production (e.g., bone marrow suppression and impaired megakaryocyte maturation).^22^ Consistent with this, Nijsten et al. demonstrated that diminished platelet production in critically ill patients correlates with poorer outcomes, potentially explaining why patients with delayed TP recovery fare worse.^16^ Second, the lower hemoglobin levels observed in our persistent SAT group, while partially attributable to hemodilution from initial fluid resuscitation, may also reflect ongoing micro-bleeding, occult blood loss, or consumptive coagulopathy exacerbated by prolonged TP.^24^

Our findings have significant clinical implications. The >3-day TP duration threshold provides clinicians with a simple, accessible tool for the early identification of high-risk septic patients. For individuals with persistent SAT, heightened vigilance for adverse outcomes is necessary, prompting consideration for intensified monitoring, aggressive management of underlying infection, and optimized organ support strategies, aligning with general principles for managing high-risk sepsis patients.^3^ Conversely, the observation that patients with transient SAT (TP resolution within 3 days) have prognoses similar to those without TP suggests that, in the presence of platelet count recovery and overall clinical improvement, unnecessary interventions or undue anxiety can be judiciously avoided. This finding, however, does not diminish the importance of dynamic platelet monitoring for all patients, as emphasized in other studies.^20^ Third, TP duration can serve as an ancillary indicator of therapeutic response. For example, persistent TP despite empirical antimicrobial therapy may signal suboptimal treatment efficacy or the presence of resistant pathogens or an undrained septic focus, necessitating a re-evaluation of the management plan, analogous to the utility of serial PCT measurements in guiding sepsis treatment.^25^

The strengths of this study include its multicenter design, incorporating large, heterogeneous sepsis patient cohorts from both Chinese and international databases, enhancing the generalizability of our findings. Furthermore, we employed robust statistical methods, including multivariable regression and PSM, to control for confounding variables, and validated our primary findings through sensitivity analyses.

The prognosis of patients with sepsis is linked to TP duration. Differentiating between persistent and transient SAT by determining whether TP duration exceeds 3 days may help direct further treatment and potentially enable early prognosis estimation. Additional research is required to validate our discoveries.

### Limitations of the study

Nevertheless, certain limitations must be acknowledged. First owing to its retrospective nature, despite adjustments for numerous known confounders, this study could not entirely preclude the influence of unmeasured or unknown confounding factors. Second, while patients with established hematological malignancies or liver cirrhosis were excluded, we could not definitively rule out the impact of newly diagnosed or undiagnosed conditions such as disseminated intravascular coagulation, thrombotic thrombocytopenic purpura, or heparin-induced TP on TP duration, although their incidence in the general sepsis population is relatively low. Third, variability in the frequency of platelet count monitoring across centers, particularly in retrospective databases, may have affected the precise calculation of TP duration, although the large sample size may partially mitigate this bias. Fourth, certain therapeutic interventions common in ICU settings, such as renal replacement therapy and extracorporeal membrane oxygenation, can independently affect platelet counts; their complex interplay with TP duration warrants further investigation. Finally, while we adjusted for the use of blood transfusions in our multivariable models, the retrospective design of this study does not permit a causal analysis of the effect of transfusions on clinical outcomes. Transfusion is both a treatment and marker of disease severity, and its complex relationship with mortality could not be fully clarified in this observational setting.

## Resource availability

### Lead contact

Requests for further information and resources should be directed to and will be fulfilled by the lead contact, Dr. Xuandong Jiang (xd_jiang1987@wmu.edu.cn).

### Materials availability

The data are available from the corresponding author on reasonable request.

### Data and code availability

• The MIMIC III dataset is a freely accessible online critical care database (https://mimic.physionet.org/).
• The R codes are available at https://github.com/fzs1412/Duration-of-Thrombocytopenia-in-Sepsis.git.
• Any additional information required to reanalyze the data reported in this paper is available from the lead contact upon request.

## Acknowledgments

We would like to thank Editage (www.editage.cn) for English language editing. This research was supported by 10.13039/501100008092Jinhua Science and Technology Bureau (grant numbers 2024-4-266and 2024-4-244).

## Author contributions

Conceptualization, W.Z. and Z.Z.; supervision, Z.Z.; data collection, X. Cai and Z.Z.; data analysis and writing – original draft, X.J.; writing – review and editing, X. Cheng and Z.Z. All authors reviewed the manuscript.

## Declaration of interests

The authors declare no competing interests.

## Declaration of generative AI and AI-assisted technologies in the writing process

During the preparation of this work, the author(s) used Google Gemini (2.5 Pro) in order to improve language and readability. After using this tool, the author(s) reviewed and edited the content as needed and take full responsibility for the content of the publication.

## STAR★Methods

### Key resources table

**Table:** 

REAGENT or RESOURCE	SOURCE	IDENTIFIER
**Deposited data**		

CMAISE1.5 Dataset	Zhang et al.^26^; Nat. Commun.	https://ngdc.cncb.ac.cn/omix/release/OMIX005601
MIMIC-III (v1.4) Dataset	Johnson et al.^27^; PhysioNet	https://doi.org/10.13026/C2XW26

**Software and algorithms**		

R (v4.1.3)	R Foundation for Statistical Computing	https://www.R-project.org/
X-tile (v3.6.1)	Rimm Lab, Yale School of Medicine	https://medicine.yale.edu/pathology/research/rimm/research/software/

### Method details

#### Study design

This retrospective, multicenter cohort study utilized data from adult patients (aged ≥18 years) with sepsis admitted to the intensive care unit (ICU). Data were sourced from Dongyang People’s Hospital (a tertiary teaching hospital in China) between December 2012 and October 2020 and two publicly available databases: the Medical Information Mart for Intensive Care III (MIMIC-III, version 1.4)^27^ and the Chinese Multi-omics Advances In Sepsis 1.5 Consortium (CMAISE1.5), a study conducted from November 2020 to December 2023 involving 35 Chinese hospitals.^26^ The study adhered to the STROBE (Strengthening the Reporting of Observational Studies in Epidemiology) guidelines (Table S1). Ethical approval was obtained from the Institutional Review Board of Dongyang People's Hospital (DRY-2022-YX-155). Due to the retrospective and observational nature of the study, the requirements of informed consent were waived by the ethics committee of Dongyang People’s Hospital. Data were de-identified prior to analysis. Author X.J. obtained access to the MIMIC-III database after completing the “Protecting Human Research Participants” online training course (Certification No. 7632299).

The inclusion criteria were as follows: first ICU admission with a diagnosis of sepsis. The exclusion criteria were as follows: age <18 years, ICU length of stay <72 hours, pre-ICU platelet count <100×10^9^/L, known hematological malignancies, liver cirrhosis, or prior splenectomy. The criterion to exclude patients with pre-existing thrombocytopenia was implemented to specifically analyze TP developing as an acute consequence of the septic event requiring ICU care.

#### Data collection

Data from Dongyang People’s Hospital were extracted using the LE9 Health electronic medical record system (Shanghai, China). Collected variables included (1) baseline demographics and clinical characteristics (age, sex, Acute Physiology and Chronic Health Evaluation [APACHE] II score, Sequential Organ Failure Assessment [SOFA] score, and pre-existing comorbidities such as hypertension and diabetes), (2) biochemical parameters (e.g., complete blood counts, blood gas analysis, liver and renal function tests, coagulation profiles), and (3) vital signs.

The primary outcome measure of the study was in-hospital mortality. Additional measures assessed during the 28 days (considering the impact of survivorship bias) included the time without ventilator support, time without ICU admission, time without hospitalization (including associated costs), and frequency of blood transfusions throughout the ICU stay. “Ventilator-free days” denotes the number of days, up to day 28, when the patient remained alive and could breathe without needing ventilator support. “ICU-free days” refers to the number of days the patient remained alive and was discharged from the ICU before the 28th day. Patients who passed away before the 28th day were regarded as having no day without ventilator support or ICU stay.^28^

#### Definitions

SAT was defined as a combination of sepsis and TP.^6^^,^^14^^,^^21^ Sepsis was identified according to the Sepsis-3 criteria, defined as a suspected infection accompanied by an acute increase of ≥2 points in the Sequential Organ Failure Assessment (SOFA) score.^29^ TP was defined as a platelet count below 100×10^9^/L during the ICU stay.^12^ Severe TP was characterized by a platelet count below 50×10^9^/L throughout the patient’s ICU stay.^30^^,^^31^

#### Classification of thrombocytopenia duration

TP duration was determined from the recorded onset and resolution times of TP during ICU stay. The duration was defined as the number of consecutive days the platelet count remained below 100×10^9^/L. An episode was considered to have resolved on the day the measured platelet count was ≥100×10^9^/L. Patients were categorized into three groups: no TP (platelet count consistently ≥100×10^9^/L throughout ICU stay), transient TP (TP duration of 1–3 days), and persistent TP (TP duration >3 days). This 3-day cutoff was based on literature review,^8^^,^^32^ the median TP duration observed in our cohorts (Dongyang: 3.2 days; MIMIC-III: 3.5 days), and clinical judgment. To assess the robustness of this cutoff, sensitivity analyses were conducted by repeating the primary multivariable logistic regression model using 2-day and 4-day thresholds in the Dongyang and MIMIC-III datasets (due to limitations in platelet recording frequency in CMAISE1.5). Additionally, the X-tile software (version 3.6.1, Yale University) was used to identify an optimal cut-point for TP duration in relation to in-hospital mortality, further evaluating the 3-day threshold (Figure S1). To further address potential survival bias from excluding patients with early mortality, we conducted an additional sensitivity analysis including all patients with an ICU stay of ≥24 hours. For patients who died between 24 and 72 hours, if their last recorded platelet count was below 100×10^9^/L, they were categorized into the persistent TP group for this analysis, based on the high likelihood that their condition would not have resolved.

### Quantification and statistical analysis

#### Data processing

Variables containing >20% missing values were removed. The missing values of variables with a loss rate <20% were replaced using multiple imputations. Missing values were handled by identifying outliers using the interquartile range.

#### Statistical analyses

R, version 4.1.3 (R Foundation for Statistical Computing, Vienna, Austria) and X-tile, version 3.6.1 (Robert  L. Camp, Yale School of Medicine, New Haven, CT, USA) were used for all analyses and calculations. Conventional descriptive statistics analysis was performed using the CBCgrps package in R.^33^ Normally distributed data were presented as means with standard deviations, whereas non-normally distributed data were presented as medians with the 25th and 75th percentiles (P25, P75). Baseline characteristics were compared across groups using analysis of variance for continuous variables and chi-square tests for categorical variables. A p-value <0.05 was considered statistically significant.

Multivariable logistic regression models were constructed to evaluate the association between TP duration categories (transient TP, persistent TP, with no TP as the reference) and in-hospital mortality. Models were adjusted for potential confounders identified from prior literature and clinical expertise, including: age, sex, APACHE II score, SOFA score, mechanical ventilation, vasopressor use, renal replacement therapy, primary site of infection, comorbidities (hypertension, diabetes, chronic obstructive pulmonary disease), admission type (surgical/non-surgical), transfusion of blood products, and use of antiplatelet agents.^5^^,^^20^ Variance Inflation Factor (VIF) was used to assess multicollinearity, with VIF <10 indicating no serious multicollinearity. In a supplementary analysis of the Dongyang cohort, we calculated the median laboratory values (platelet count, hemoglobin, and INR/PT) on the day of the first transfusion for each respective blood component to determine the clinical triggers for transfusion.

To further control for selection bias and potential confounders, propensity score matching (PSM) was performed. Separate PSM models were used to compare the transient TP group with the no TP group and the persistent TP group with the no TP group. This analytical strategy was chosen to specifically isolate the independent prognostic impact of each TP subtype against a common, clinically relevant reference group. Propensity scores were estimated using logistic regression incorporating the same covariates listed above. One-to-one nearest-neighbor matching with a caliper of 0.2 standard deviations of the logit of the propensity score was implemented. Standardized mean differences (SMDs) were calculated post-matching to assess balance, with an SMD <0.1 considered indicative of negligible imbalance. Kaplan-Meier survival curves were generated to depict in-hospital survival according to TP duration groups, and differences were assessed using the log-rank test.
