# Supplementary material for: Prognostic impact of persistent versus transient sepsis-associated thrombocytopenia in multicohort data
Source: iScience. 2025 Nov 11;28(12):114023. doi: 10.1016/j.isci.2025.114023 (PMC12682006; doi:10.1016/j.isci.2025.114023)
Supplement: Document S1. Figures S1 and S2 and Tables S1–S6 [file mmc1.pdf]

iScience, Volume *28*

Supplemental information

Prognostic impact of persistent versus transient sepsis-associated thrombocytopenia in multicohort data

Weimin Zhang, Xuping Cheng, Xufeng Cai, Zhongheng Zhang, and Xuandong Jiang

Table S1: STROBE Statement—checklist of items that should be included in reports of observational studies

	Item No.	Recommendation	Page No.	Relevant text from manuscript
**Title and abstract**	1	(*a*) Indicate the study's design with a commonly used term in the title or the abstract	1	Title
		(*b*) Provide in the abstract an informative and balanced summary of what was done and what was found	1	SUMMARY
**Introduction**				
Background/rationale	2	Explain the scientific background and rationale for the investigation being reported	1-2	Introduction
Objectives	3	State specific objectives, including any prespecified hypotheses	2	Introduction
**Methods**				
Study design	4	Present key elements of study design early in the paper	15	Study Design
Setting	5	Describe the setting, locations, and relevant dates, including periods of recruitment, exposure, follow-up, and data collection	15-16	Study Design and Data collection
Participants	6	(*a*) *Cohort study*—Give the eligibility criteria, and the sources and methods of selection of participants. Describe methods of follow-up<br>*Case-control study*—Give the eligibility criteria, and the sources and methods of case ascertainment and control selection. Give the rationale for the choice of cases and controls<br>*Cross-sectional study*—Give the eligibility criteria, and the sources and methods of selection of participants	15-16	Study Design and Data collection
		(*b*) *Cohort study*—For matched studies, give matching criteria and number of exposed and unexposed<br>*Case-control study*—For matched studies, give matching criteria and the number of controls per case		
Variables	7	Clearly define all outcomes, exposures, predictors, potential confounders, and effect modifiers. Give diagnostic criteria, if applicable	15	Data collection and Definitions
Data sources/ measurement	8*	For each variable of interest, give sources of data and details of methods of assessment (measurement). Describe comparability of assessment methods if there is more than one group	15-16	Data collection
Bias	9	Describe any efforts to address potential sources of bias	NA	
Study size	10	Explain how the study size was arrived at	NA	
Quantitative variables	11	Explain how quantitative variables were handled in the analyses. If applicable, describe which groupings were chosen and why	16	Statistical Analyses

Statistical methods	12	(*a*) Describe all statistical methods, including those used to control for confounding	16-17	Statistical Analyses
		(*b*) Describe any methods used to examine subgroups and interactions	17	Statistical Analyses
		(*c*) Explain how missing data were addressed	16	Data Processing
		(*d*) *Cohort study*—If applicable, explain how loss to follow-up was addressed<br>*Case-control study*—If applicable, explain how matching of cases and controls was addressed<br>*Cross-sectional study*—If applicable, describe analytical methods taking account of sampling strategy	NA	
		(*e*) Describe any sensitivity analyses	17	Statistical Analyses
**Results**				
Participants	13*	(a) Report numbers of individuals at each stage of study—eg numbers potentially eligible, examined for eligibility, confirmed eligible, included in the study, completing follow-up, and analysed	2	RESULTS
		(b) Give reasons for non-participation at each stage	2	RESULTS
		(c) Consider use of a flow diagram		Figure 1
Descriptive data	14*	(a) Give characteristics of study participants (eg demographic, clinical, social) and information on exposures and potential confounders		Table 3,S4
		(b) Indicate number of participants with missing data for each variable of interest	NA	
		(c) *Cohort study*—Summarise follow-up time (eg, average and total amount)	NA	
Outcome data	15*	*Cohort study*—Report numbers of outcome events or summary measures over time	3	RESULTS
		Case-control study—Report numbers in each exposure category, or summary measures of exposure		
		Cross-sectional study—Report numbers of outcome events or summary measures		
Main results	16	(*a*) Give unadjusted estimates and, if applicable, confounder-adjusted estimates and their precision (eg, 95% confidence interval). Make clear which confounders were adjusted for and why they were included	3	Figure 2
		(*b*) Report category boundaries when continuous variables were categorized	2-3	Table 1-2
		(*c*) If relevant, consider translating estimates of relative risk into absolute risk for a meaningful time period		
Other analyses	17	Report other analyses done—eg analyses of subgroups and interactions, and sensitivity analyses	3	TableS1,4-6
**Discussion**				
Key results	18	Summarise key results with reference to study objectives	3-4	DISCUSSION
Limitations	19	Discuss limitations of the study, taking into account sources of potential bias or imprecision. Discuss both direction and magnitude of any potential bias	5	DISCUSSION

Interpretation	20	Give a cautious overall interpretation of results considering objectives, limitations, multiplicity of analyses, results from similar studies, and other relevant evidence	3-4	DISCUSSION
Generalisability	21	Discuss the generalisability (external validity) of the study results	5	DISCUSSION
**Other information**				
Funding	22	Give the source of funding and the role of the funders for the present study and, if applicable, for the original study on which the present article is based	5	Funding

*Give information separately for cases and controls in case-control studies and, if applicable, for exposed and unexposed groups in cohort and cross-sectional studies.

Note: An Explanation and Elaboration article discusses each checklist item and gives methodological background and published examples of transparent reporting. The STROBE checklist is best used in conjunction with this article (freely available on the Web sites of PLoS Medicine at http://www.plosmedicine.org/, Annals of Internal Medicine at http://www.annals.org/, and Epidemiology at http://www.epidem.com/). Information on the STROBE Initiative is available at www.strobe-statement.org.

Tbale S2: Comparisons of baseline characteristics and outcomes between patients with no thrombocytopenia and thrombocytopenia in MIMIC Ⅲ.

Variables	Total (N = 5,473)	No TP (n = 3,927)	TP 1-3 days (n = 690)	TP > 3days (n = 856)	*p*-value
Age, (years)	66.4 ± 16.7	66.9 ± 16.6	66 ± 17	64.3 ± 16.5	< 0.001
Sex, men (%)	2941 (53.7)	2088 (53.2)	391 (56.7)	462 (54)	< 0.001
Hypertension (%)	2080 (38)	1564 (39.8)	248 (35.9)	268 (31.3)	< 0.001
Diabetes (%)	1443 (26.4)	1083 (27.6)	177 (25.7)	183 (21.4)	< 0.001
COPD (%)	264 (4.8)	225 (5.7)	17 (2.5)	22 (2.6)	< 0.001
Admission type (%)					< 0.001
ELECTIVE	494 (9)	325 (8.3)	84 (12.2)	85 (9.9)	
EMERGENCY	4787 (87.5)	3483 (88.7)	578 (83.8)	726 (84.8)	
URGENT	192 (3.5)	119 (3)	28 (4.1)	45 (5.3)	
RRT (%)	322 (5.9)	114 (2.9)	41 (5.9)	167 (19.5)	< 0.001
SOFA score	6.1 ± 3.2	5.3 ± 2.7	7 ± 3.3	8.7 ± 3.7	< 0.001
Vasopressor use (%)	2310 (42.2)	1454 (37)	335 (48.6)	521 (60.9)	< 0.001
Duration of TP, (days)	0 (0, 0)	0 (0, 0)	1.01 (0, 2.04)	6.59 (4.35, 12.86)	< 0.001
Severe TP (%)	524 (9.6)	0 (0)	59 (8.6)	465 (54.3)	< 0.001
White blood cell, (×109/L)	13.1 ± 6	13.6 ± 5.7	12.4 ± 6	11.6 ± 6.8	< 0.001
Red blood cell, (×109/L)	3.6 ± 0.6	3.6 ± 0.6	3.5 ± 0.7	3.3 ± 0.7	< 0.001

Platelet count , (×109/L)	222.8 ± 123.4	258.8 ± 118.7	147.1 ± 76.3	118.5 ± 81.2	< 0.001
Prothrombin time, (s)	14.6 (13.4, 16.9)	14.2 (13.2, 16.2)	15.2 (13.7, 17.3)	16.25 (14.3, 19.32)	< 0.001
pH	7.3 ± 0.1	7.4 ± 0.1	7.3 ± 0.1	7.3 ± 0.1	
PCO2, (mmHg)	42.5 ± 10.7	43.4 ± 10.7	41.1 ± 10	39.6 ± 10.3	< 0.001
PO2, (mmHg)	171.7 ± 126	167.2 ± 121.4	194.9 ± 138.2	173.2 ± 134.5	< 0.001
Bicarbonate, (mmol/L)	22.6 ± 4.7	23.3 ± 4.5	21.7 ± 4.5	20.5 ± 5.1	< 0.001
Lactate, (mmol/L)	1.9 (1.3, 3.1)	1.8 (1.2, 2.7)	2.31 (1.5, 3.8)	2.6 (1.6, 4.4)	< 0.001
Creatinine, (mmol/L)	1.1 (0.8, 1.7)	1 (0.7, 1.6)	1 (0.8, 1.67)	1.3 (0.8, 2.2)	< 0.001
Urea level, (mmol/L)	23 (15, 39)	22 (14, 37)	22 (15, 36)	28 (17, 48)	< 0.001
ICU free days	18.63 (7.82, 23.21)	20.06 (7.82, 23.62)	16.82 (7.82, 22.01)	9.24 (0, 20.26)	< 0.001
Ventilator free days	0 (0, 20.5)	0 (0, 24.49)	0 (0, 7.98)	0 (0, 0)	< 0.001
Hospital free days	8.96 (0, 16.71)	10.92 (0, 17.58)	6.79 (0, 15.11)	0 (0, 11.21)	< 0.001
Hospital mortality (%)	1231 (22.5)	736 (18.7)	172 (24.9)	323 (37.7)	< 0.001

Abbreviations: MIMIC, Medical Information Mart for Intensive Care; TP, thrombocytopenia; COPD, Chronic obstructive pulmonary disease; ICU, intensive care unit; RRT, Renal replacement therapy; SOFA, Sepsis-related Organ Failure Assessment; PCO2, Partial Pressure of Carbon Dioxide; PO2, Partial Pressure of Oxygen.

Table S3. Comparisons of baseline characteristics and outcomes between patients with no thrombocytopenia and thrombocytopenia in CMAISE

**Variables**	**Total (N = 1103)**	**No TP (n = 497)**	**TP 1-3 days (n = 173)**	**TP > 3days (n = 433)**	***p*-value**
Age, (years)	67.9 ± 15.7	68.5 ± 16	67.8 ± 17.3	67.2 ± 14.7	0.501
Sex, men (%)	690 (62.6)	325 (65.4)	101 (58.4)	264 (61)	0.177
Diabetes (%)	301 (27.3)	145 (29.2)	34 (19.7)	122 (28.2)	0.046
Hypertension (%)	488 (44.2)	239 (48.1)	71 (41)	178 (41.1)	0.066
Myocardial infarction (%)	41 (3.7)	26 (5.2)	3 (1.7)	12 (2.8)	0.046
Cardiac failure (%)	126 (11.4)	72 (14.5)	15 (8.7)	39 (9)	0.015
SOFA score	8.6 ± 3.6	6.8 ± 3	8.9 ± 3.6	10.4 ± 3.3	< 0.001
Fluid intake (ml)	2737 (1777.5, 4070.5)	2300 (1474, 3265)	2890 (1820, 4321)	3345 (2162, 4889)	< 0.001
Fluid output (ml)	1400 (700, 2377.5)	1436 (745, 2230)	1350 (600, 2400)	1420 (700, 2580)	0.749
Urine output (ml)	1000 (400, 1800)	1000 (500, 1780)	870 (300, 1750)	960 (356, 1850)	0.51
Maximum heart rate (bpm)	118.7 ± 26	114.6 ± 25.4	120.4 ± 27.8	122.6 ± 25.2	< 0.001
Minimum heart rate (bpm)	84.1 ± 19.4	81.4 ± 18.9	83.3 ± 20.1	87.4 ± 19.3	< 0.001
Maximum mean arterial pressure (mmHg)	98.8 ± 17.4	100.3 ± 17.9	96.7 ± 15.3	97.9 ± 17.5	0.024
Minimum mean arterial pressure (mmHg)	65.9 ± 12.9	67.6 ± 13.3	64.8 ± 13.3	64.4 ± 12	< 0.001

White blood cell, (×109/L)	12.2 (7.6, 18)	12.7 (9.1, 18.5)	11.6 (6.5, 17)	11.4 (6.5, 17.5)	< 0.001
Hematocrit (%)	34 ± 9.6	34.1 ± 9.4	35 ± 12	33.5 ± 8.8	0.187
Platelet count , (×109/L)	158.7 ± 100.7	226 ± 96.3	143.4 ± 69.5	87.6 ± 54.2	< 0.001
pH	7.4 (7.3, 7.4)	7.4 (7.3, 7.4)	7.4 (7.3, 7.4)	7.4 (7.3, 7.4)	0.055
PCO2, (mmHg)	32.8 (27.8, 39)	35 (29.2, 41.3)	32.6 (28.7, 38.7)	30.8 (26.3, 36)	< 0.001
PO2, (mmHg)	93.3 (73.6, 123)	93.1 (73.6, 123)	94 (75.7, 126)	93.4 (73, 120)	0.618
Lactate, (mmol/L)	2.5 (1.5, 4.4)	2 (1.4, 3.2)	2.3 (1.4, 4)	3.3 (2.1, 5.6)	< 0.001
Creatinine, (μmol/L)	124 (79.6, 210.6)	101.6 (71, 171.9)	124.8 (80, 227.1)	145.6 (98, 239)	< 0.001
C-reactive protein (mg/L)	133.3 (63.2, 200)	112 (55, 193.7)	129.2 (54.8, 220)	156.8 (81, 224.6)	< 0.001
Hospital mortality (%)	129 (11.7)	39 (7.8)	24 (13.9)	66 (15.2)	0.001
Ventilator free days	24 (17, 28)	26 (20, 28)	24 (16, 28)	23 (14, 28)	< 0.001
RRT free days	28 (25, 28)	28 (28, 28)	28 (24, 28)	28 (23, 28)	< 0.001
Hospital free days	12.4 (0, 18.6)	13.9 (2.4, 19)	11.1 (0, 17.2)	11.4 (0, 18)	0.007

Abbreviations: CMAISE, Chinese Multi-omics Advances In Sepsis; TP, thrombocytopenia; COPD, Chronic obstructive pulmonary disease; ICU, intensive care unit; RRT, Renal replacement therapy; SOFA, Sepsis-related Organ Failure Assessment; PCO2, Partial Pressure of Carbon Dioxide; PO2, Partial Pressure of Oxygen.

Table S4. Multivariable logistic regression analysis of hospital mortality risk associated with thrombocytopenia duration using a 2-day cutoff

	Adjusted OR (95% CI)	
Duration of thrombocytopenia	Dongyang	MIMIC Ⅲ
None	1.00 Reference	1.00 Reference
1–2 days	1.33 (0.91–1.93)	1.13 (0.89–1.43)
≥2 days	1.79 (1.29–2.47)	1.64 (1.37–1.96)

Abbreviations: 95% CI, 95% confidence interval; OR: odds ratio; Dongyang model: adjusted for age, sex, Acute Physiology and Chronic Health Evaluation II score, mechanical ventilation, chronic obstructive pulmonary disease, pH, lactate, surgery, renal replacement therapy, antiplatelet drug use, and prothrombin time. MIMIC-III model: adjusted for age, sex, Sequential Organ Failure Assessment score, hypertension, chronic obstructive pulmonary disease, vasopressor use, prothrombin time, creatinine, pH, lactate, and surgery.

Table S5. Multivariable logistic regression analysis of hospital mortality risk associated with thrombocytopenia duration using a 4-day cutoff

	Adjusted OR (95% CI)	
Duration of thrombocytopenia	Dongyang	MIMIC Ⅲ
None	1.00 Reference	1.00 Reference
1–4 days	1.36 (1.00–1.84)	1.22 (1.01–1.48)
≥4 days	1.98 (1.38–2.84)	1.80 (1.47–2.21)

Abbreviations: 95% CI, 95% confidence interval; OR: odds ratio; MIMIC: Medical Information Mart for Intensive Care; Dongyang model: adjusted for age, sex, APACHE II score, mechanical ventilation, chronic obstructive pulmonary disease, pH, lactate, surgery, renal replacement therapy, antiplatelet drug use, and prothrombin time. MIMIC-III model: adjusted for age, sex, SOFA score, hypertension, chronic obstructive pulmonary disease, vasopressor use, prothrombin time, creatinine, pH, lactate, and surgery.

Table S6. Sensitivity analysis: multivariable logistic regression of hospital mortality risk in patients with ICU stay ≥24 hours

	Adjusted OR (95% CI)		
Duration of thrombocytopenia	Dongyang	MIMIC Ⅲ	CMAISE
None	1.00 Reference	1.00 Reference	1.00 Reference
1–3 days	1.35 (0.95–1.90)	1.32 (0.92–1.87)	1.29 (0.69–2.35)
>3 days	1.86 (1.33–2.59)	1.41 (1.21–2.64)	1.73 (1.01–2.99)

Abbreviations: 95% CI, 95% confidence interval; OR: odds ratio; Dongyang model: adjusted for age, sex, Acute Physiology and Chronic Health Evaluation II score, mechanical ventilation, chronic obstructive pulmonary disease, pH, lactate, surgery, renal replacement therapy, antiplatelet drug use, transfusion of blood products, and prothrombin time. MIMIC-III model: adjusted for age, sex, Sequential Organ Failure Assessment score,

hypertension, chronic obstructive pulmonary disease, vasopressor use, prothrombin time, creatinine, pH, lactate, and surgery; CMAISE model: adjusted for age, sex, hypertension, diabetes, SOFA score, lactate, pH, renal replacement therapy, platelet count, mechanical ventilation, and chronic obstructive pulmonary disease.

Figure S1 X-tile analysis of the duration of thrombocytopenia for hospital mortality

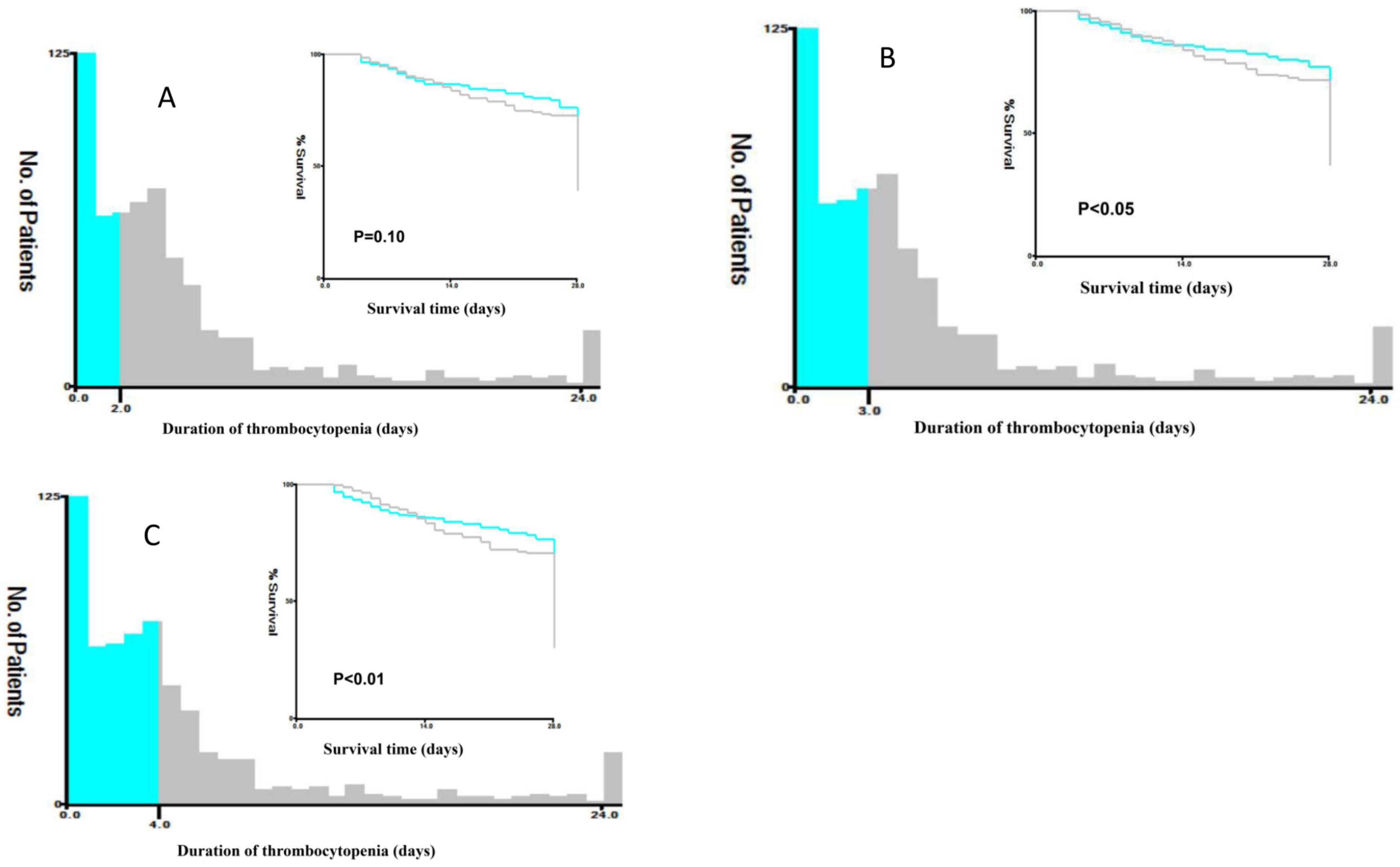

A: The cut-off values of duration of thrombocytopenia is 2 days. B: The cut-off values of duration of thrombocytopenia is 3 days. C: The cut-off values of duration of thrombocytopenia is 4 days.

Figure S2 Transfusion threshoids for blood components in the Dongyang cohort

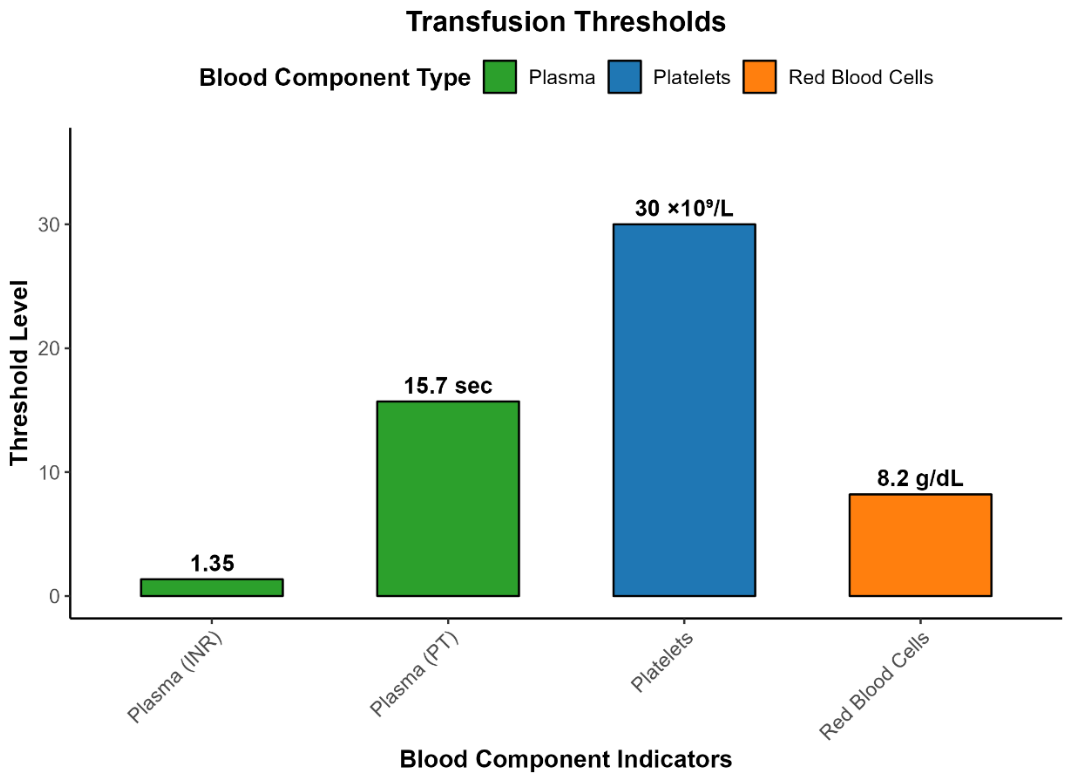

The bar chart displays the median laboratory values that served as transfusion triggers. The indicators shown are International Normalized Ratio (INR) and

Prothrombin Time (PT) for plasma, platelet count for platelets, and hemoglobin level for red blood cells (RBC).
